# Plasma biomarkers of amyloid, tau, axonal, and neuroinflammation pathologies in dementia with Lewy bodies

**DOI:** 10.1186/s13195-024-01502-y

**Published:** 2024-07-03

**Authors:** Agathe Vrillon, Olivier Bousiges, Karl Götze, Catherine Demuynck, Candice Muller, Alix Ravier, Benoît Schorr, Nathalie Philippi, Claire Hourregue, Emmanuel Cognat, Julien Dumurgier, Matthieu Lilamand, Benjamin Cretin, Frédéric Blanc, Claire Paquet

**Affiliations:** 1 AP-HP Nord, Cognitive Neurology Center Hôpital Lariboisière-Fernand Widal, Université Paris Cité, 200 rue du Faubourg Saint-Denis, Paris, 75010 France; 2 Université Paris Cité, INSERM, UMRS 1144, Paris, France; 3 University of California San Francisco, San Francisco, USA; 4 Laboratory of Biochemistry and Molecular Biology, University Hospital of Strasbourg, Strasbourg, France; 5 University of Strasbourg and CNRS, ICube laboratory UMR 7357 and FMTS (Fédération de Médecine Translationnelle de Strasbourg), team IMIS Strasbourg, Strasbourg, France; 6 CM2R (Memory Resource and Research Centre), Service of Gerontology Mobile-Neuro-Psy-Research, Geriatrics Department, University Hospital of Strasbourg, Strasbourg, France; 7 Neuropsychology unit, Service of Neurology Strasbourg, University Hospital of Strasbourg, Strasbourg, France

**Keywords:** Dementia with Lewy bodies, Alzheimer’s disease, Plasma biomarkers, Amyloid pathology

## Abstract

**Background:**

Increasing evidence supports the use of plasma biomarkers of amyloid, tau, neurodegeneration, and neuroinflammation for diagnosis of dementia. However, their performance for positive and differential diagnosis of dementia with Lewy bodies (DLB) in clinical settings is still uncertain.

**Methods:**

We conducted a retrospective biomarker study in two tertiary memory centers, Paris Lariboisière and CM2RR Strasbourg, France, enrolling patients with DLB (*n* = 104), Alzheimer’s disease (AD, *n* = 76), and neurological controls (NC, *n* = 27). Measured biomarkers included plasma Aβ40/Aβ42 ratio, p-tau181, NfL, and GFAP using SIMOA and plasma YKL-40 and sTREM2 using ELISA. DLB patients with available CSF analysis (*n* = 90) were stratified according to their CSF Aβ profile.

**Results:**

DLB patients displayed modified plasma Aβ ratio, p-tau181, and GFAP levels compared with NC and modified plasma Aβ ratio, p-tau181, GFAP, NfL, and sTREM2 levels compared with AD patients. Plasma p-tau181 best differentiated DLB from AD patients (ROC analysis, area under the curve [AUC] = 0.80) and NC (AUC = 0.78), and combining biomarkers did not improve diagnosis performance. Plasma p-tau181 was the best standalone biomarker to differentiate amyloid-positive from amyloid-negative DLB cases (AUC = 0.75) and was associated with cognitive status in the DLB group. Combining plasma Aβ ratio, p-tau181 and NfL increased performance to identify amyloid copathology (AUC = 0.79). Principal component analysis identified different segregation patterns of biomarkers in the DLB and AD groups.

**Conclusions:**

Amyloid, tau, neurodegeneration and neuroinflammation plasma biomarkers are modified in DLB, albeit with moderate diagnosis performance. Plasma p-tau181 can contribute to identify Aβ copathology in DLB.

**Supplementary Information:**

The online version contains supplementary material available at 10.1186/s13195-024-01502-y.

## Introduction

Dementia with Lewy bodies (DLB) is evaluated to be the third cause of dementia after Alzheimer’s disease (AD) and vascular dementia. DLB is characterized by a poorer prognosis, higher healthcare costs and caregiver burden, and a greater impact on quality of life compared with AD [1, 2]. Yet, therapeutic research in DLB has long remained a poorly invested field, and there are no disease-specific treatments currently approved by the European Medicines Agency. However, this field has known some recent expansion, with more than 30 ongoing trials in 2022, including evaluations of disease modifier molecules [3]. In this context, achieving early and reliable diagnosis should be a priority.

Patients with DLB present with a wide range of cognitive, psychiatric, motor, and dysautonomic symptoms that can vary in-between patients and over time within individuals. The currently used diagnosis criteria, defined by McKeith et al., include imaging and electrophysiological supportive biomarkers in association with core clinical features [4]. However, no specific markers of pathology are included.

DLB is characterized by the presence of Lewy bodies formed primarily of alpha-synuclein. Amyloid-beta (Aβ) plaques, and to a lesser extent hyperphosphorylated tau (p-tau) tangles, may also be present [5, 6]. Cerebral and peripheral inflammation has been demonstrated to be contributing mechanisms, especially at the early stages of the disease [7]. Specific blood-based assays would benefit diagnostic in clinical practice and facilitate clinical trials, as non-invasive and scalable. Real-time quaking-induced conversion (RT-QuIC) assays are showing high sensitivity and specificity to detect alpha-synuclein in cerebrospinal fluid (CSF) in DLB and Parkinson’s disease (PD [8–10]. However, they still require further validation, especially in plasma [11]. Interestingly, number of amyloid and tau plasma biomarkers, as well as axonal and neuroinflammatory markers have been studied in DLB and appear modified. Plasma p-tau has demonstrated good accuracy to identify an AD copathology commonly present in DLB [12–14]. Indeed, more than half of patients with DLB demonstrate coexisting amyloid lesions, that impact clinical presentation and disease progression [5, 6]. Several plasma p-tau isoforms, including plasma p-tau181, p-tau231, and p-tau217, were reported to be higher in DLB patients with CSF or PET evidence of amyloid copathology [12, 14, 15]. Plasma NfL and glial fibrillary acidic protein (GFAP) levels were found to be higher in Lewy body dementia (DLB and Parkinson’s disease dementia) compared to controls but lower compared to AD [16, 17]. Glial markers, including soluble triggering receptor expressed on myeloid cells 2 (sTREM2) and human chitinase-3 (YKL-40) have shown inconsistent modifications in DLB, both in CSF and in plasma, but nevertheless suggesting different glial activation patterns than in AD [18, 19].

While being nonspecific and considering the presence of neuroinflammation and the possible co-existence of AD pathology, our question is could those existing plasma biomarkers be useful in DLB diagnosis? The aim of this study was to investigate 6 plasma biomarkers, as standalone markers or in combinations to evaluate their usefulness in DLB diagnosis, in comparison to AD patients and control individuals.

## Methods

### Cohorts

In a cross-sectional retrospective bicentric study, we analyzed samples from the Cognitive Neurology Center, Lariboisière Hospital, Université Paris Cité, Paris, France, and the Memory Clinic (CM2R) of Strasbourg, Alsace, France from 2012 to 2021. The cohort consecutively included 27 neurological control subjects (Paris: *n* = 19, Strasbourg: *n* = 8), 104 patients with DLB (Paris: *n* = 56, Strasbourg: *n* = 48), and 76 patients with AD (Paris: *n* = 71, Strasbourg: *n* = 5).

All included subjects underwent comprehensive neurological examination, neuropsychological evaluation, and brain imaging. All controls and AD subjects underwent lumbar puncture as well as 87% (90/104) of DLB patients. Consensus diagnoses were validated after a multidisciplinary review of cases, by neurologists, neuropsychologists, neuroradiologists, and biologists. All DLB patients fulfilled the most recent revised diagnosis criteria for probable DLB, established by McKeith et al. [4]. Diagnosis of AD, including MCI and dementia patients, was made according to Albert’s et al. criteria and Dubois et al. criteria [20, 21]. All patients with AD displayed a CSF amyloid-positive profile. Neurological controls were individuals seen at the clinic but for whom, no evidence of neurocognitive disease was found; they presented with normative or sub-normative cognitive testing, normal morphological brain imaging, and normal CSF profile. Those subjects included some participants in observational research studies and individuals with subjective cognitive complaints in the context of minor depressive symptoms, sleep disorders, chronic pain or chronic fatigue, or systemic disorders. The cognitive status was assessed with the mini-mental state evaluation (MMSE).

### Plasma biomarkers measurements

Plasma samples were obtained through venipuncture, in the morning in fasting conditions, on the same day as CSF uptake. After a 20-minute centrifugation at 1,900 x g within 4 h, plasma was aliquoted in polypropylene tubes and stored at − 80 °C until analysis.

All analyses were performed in Inserm U1144, Université Paris Cité, Paris, France.

Plasma Aβ40, Aβ42, NfL, and GFAP were measured with the Simoa Neurology 4-plex E kit from Quanterix®. Plasma p-tau181 was measured with the Simoa p-tau181 Advantage V2 assays also from Quanterix®. Samples were analyzed blinded in singlicate. All samples were above the threshold of quantification. All intra and inter coefficients of variations were below 10% (Aβ40: intraplate CV = 2.7%, interplate CV = 4.2%; Aβ42: intraplate CV = 1.2%, interplate CV;=3.4%; GFAP, intra plate CV = 9.9%, interplate CV = 6.0%; NfL, intraplate CV = 6.1%, interplate CV = 3.0%; p-tau181: intraplate CV = 9.5%, inter plate = 5.3% ).

Plasma sTREM2 and YKL-40 levels were measured using commercial ELISA kits from R&D System (respectively, #DY1828-05 and #DY2599), both validated for plasma measurements. Samples were run blinded in duplicates. Intra- and inter-coefficients of variations were respectively 7.1% and 10.7% for sTREM2 and 5.1% and 6.6% for plasma YKL-40.

Apolipoprotein E (APOE) genotype was determined through analysis of 2 single nucleotide variations (formerly single nucleotide polymorphisms, rs429358, and rs7412) using established standard polymerase chain reaction as described in Dumurgier et al. [22].

### CSF AD biomarkers

CSF AD biomarkers analysis was available for all control subjects, AD patients, and 87% of DLB patients CSF was collected by standard lumbar puncture procedure. CSF samples were collected in polypropylene tubes for CSF AD biomarker measurements. In Paris cohort, CSF Aβ42, Aβ40, t-tau, and p-tau181 were measured by different assays across time, including Innotest Fujirebio® (*n* = 30) and Elecsys Roche® for Aβ42, p-tau181 and t-tau and Innotest Fujirebio® for Aβ40 (*n* = 101). In Strasbourg cohort, CSF Aβ42, Aβ40, t-Tau, and p-tau181 were measured with Innotest Fujirebio® for the largest part (*n* = 58) and with Lumipulse Fujirebio® for *n* = 3 subjects. Assays and cut-offs are detailed in Supp. Table 1.

Patients with available CSF AD biomarkers were classified according to the AT(N) classification [23].

### Statistical analysis

Statistical analyses were conducted using SPSS® version 29.0 (IBM statistics) and Graphpad® Prism version 9.0 (GraphPad, San Diego, CA, USA). Continuous nonparametric data are presented as median (interquartile range) and parametric data as mean (standard deviation).

Age, MMSE, and levels of education were compared between diagnostic groups using Kruskall Wallis test and sex and APOE ɛ4 carriership using Chi-2 test. Plasma biomarker levels did not display a normal distribution and were log-transformed before analysis. Association of the biomarkers with age, sex, and APOE ɛ4 carriership was studied unadjusted (with Spearman’s correlation for age and chi-2 test for sex and APOE status) then adjusting for age, sex, and APOE status using linear regression.

One-way analysis of variance adjusted on age and sex with Tukey’s LSD post hoc analysis, adjusting for multiple comparisons, was used to assess biomarker level differences between the diagnostic groups. Effect sizes were estimated with *Cohen’s d*.

Receiver operating characteristic (ROC) analysis with area under the curve (AUC) calculation was obtained by performing logistic regression, including age and sex as covariates, to study the diagnosis performance of the plasma biomarkers. Combination of biomarkers were studied using logistic binary regression, in a stepwise backward elimination strategy based on Wald to identify the best combination for differentiation between diagnosis groups. Areas under the curve (AUCs) were compared with the Akaike information criterion (AIC).

The AD group was analyzed as two groups, AD-MCI and AD dementia, in secondary analyses.

To explore the association of our plasma biomarkers with amyloid copathology, we stratified DLB patients according to the AT scheme, dichotomizing on the A status defined by CSF Aβ42/Aβ40 ratio (A + versus A-), then by AT status (A + T + versus A-T-). The differences in plasma biomarker levels were studied using one-way analysis of variance adjusting on age and sex. Effect sizes were estimated using *eta squared* η^2^.

MMSE scores were transformed into the square root of the number of errors (√[30-MMSE]) to ensure normalcy of distribution [24]. The association of biomarkers with MMSE was studied using Spearman’s correlation and with linear regression adjusting on age, sex, and level of education.

Principal component analysis (PCA) was performed in the DLB and AD groups to explore the pattern of association between the different biomarkers. Outlier values, defined by a value > mean ± 3SD, were excluded for each biomarker before analysis. Kaiser–Meyer–Olkin Measure of Sampling Adequacy test and Bartlett’s Test of Sphericity were used to evaluate the suitability of the dataset. The number of components was determined by the number of eigenvalues greater than one. Variables with a loading factor > 0.4 or < − 0.4 were regarded as representative of the component.

## Results

The overall cohort’s demographic characteristics and plasma biomarker levels are presented in Table 1, and by center in Supp. Table 2. DLB and AD patients were significantly older than control individuals (*P* < 0.001). We observed a higher percentage of males in the DLB group than in the AD and control groups (*P* = 0.037). The AD group displayed more frequent APOE ɛ4 carriership than the DLB and NC groups (*P* < 0.001). In the AD group, 97% (*n* = 74) displayed a CSF A + T + profile and 3% (*n* = 2) an A + T- profile. As a sensitivity analysis, the main analyses have been reproduced after the exclusion of the A + T- subjects and yielded similar results (Suppl. Figure 1). Additionally, the characteristics of AD-MCI and AD-dementia groups are presented in Supp. Table 3.

**Table 1:** Cohort characteristics and plasma biomarkers levels

	NC	DLB	AD	
n total = 207	*n* = 27	*n* = 104	*n* = 76	*P*-value
Age, year	61.3 [10.0]	71.2 [11.4]	72.1 [11.8]	**< 0.001** ^**a**^
Sex, male	41% (11)	59% (61)	41% (31)	**0.037** ^**b**^
APOE ɛ4 carriership	32% (8/25)	39% (31/79)	68% (47/69)	**< 0.001** ^**b**^
MMSE	28.5 [3.00]	25.0 [5.75]	19.0 [5.50]	**< 0.001** ^**a**^
Level of education, year	15.0 [5.00]	11.0 [6.00]	11.0 [6.00]	**0.002** ^**a**^
**CSF biomarkers, ATN**				**< 0.001** ^**a**^
Available (n)	100% (27)	87% (90)	100% (76)	
A-T-	100% (27)	59% (53)	0% (0)	
A + T-	0% (0)	17% (15)	3% (2)	
A + T+	0% (0)	12% (11)	97% (74)	
A-T+	0% (0)	12% (11)	0% (0)	
**Plasma biomarkers**				
Plasma Aβ40/Aβ42 ratio	0.0646 [0.0140]	0.0589 [0.0181]	0.0534 [0.0154]	**< 0.001**
Plasma GFAP, pg/mL	59.4 [35.3]	107 [81.5]	157 [112]	**< 0.001**
Plasma NfL, pg/mL	17.2 [7.95]	21.5 [14.0]	25.3 [12.7]	**< 0.001**
Plasma p-tau181, pg/mL	1.62 [0.788]	2.03 [1.52]	3.81 [1.79]	**< 0.001**
Plasma sTREM2, pg/mL	444 [274]	629 [397]	518 [415]	**0.029**
Plasma YKL-40, ng/mL	73.7 [75.3]	87.7 [103]	79.2 [62.4]	0.091

Continuous variables are presented as median [IQR] and categorical data as number (%)

^a^Age, MMSE scores, and level of education were compared between groups using the Kruskall-Wallis test. ^b^APOE ɛ4 carriership frequency and ATN profiles were compared between groups using Chi2 test. In-between groups comparison of plasma biomarker levels was performed using one-way ANCOVA adjusted on age and sex

Abbreviations: AD, Alzheimer’s disease; APOE, apolipoprotein E, CSF, cerebrospinal fluid, DLB, dementia with Lewy bodies; GFAP, glial fibrillary acidic protein; NC, neurological controls; MMSE, mini-mental state examination; NfL, neurofilament light chain; p-tau181, tau phosphorylated at serine 181; sTREM2, soluble triggering receptor expressed myeloid cells 2

Associations with age, sex, and APOE status are detailed in Supp. Table 4. In the whole cohort, all plasma biomarkers were associated with age (β = 0.236–0.538, *P* ≤  0.002) except for Aβ ratio (β=-0.042, *P* = 0.593) after adjustment on sex and APOE ɛ4 carriership. Plasma GFAP and YKL-40 levels were higher in females after adjustment on age and APOE status (GFAP: β = 0.258, *P* < 0.001; YKL-40, β = 0.198, *P* = 0.008). Plasma Aβ ratio, p-tau181, and sTREM2 levels were associated with APOE ɛ4 carriership in adjusted analysis (β = 0.165-0.228, *P* ≤ 0.028). Focusing on the DLB group, after adjustment for covariates, we found positive associations between age and plasma p-tau181, NfL, GFAP, and YKL-40 levels (β = 0.247–0.521, *P*  ≤0.030) and between female sex and plasma GFAP levels (β = 0.259, *P* = 0.008). No association was found between any plasma marker and ApoE4 carriership, after adjustment for age and sex in the DLB group.

Correlations between biomarkers are displayed in Supp. Figure 2. Focusing on DLB patients, plasma GFAP, p-tau181, and NfL showed significant associations (*r* = 0.341–0.560, *P* < 0.0001 overall). Plasma YKL-40 and sTREM2 were significantly associated (*r* = 0.284, *P* = 0.003), as well as with plasma NfL (*r* = 0.406, *P* < 0.000 both). Plasma GFAP was the only marker significantly associated with the plasma Aβ ratio (*r*=-0.325, *P* < 0.0001), though there was a tendency to association between the Aβ ratio and p-tau181 (*r*=-0.185, *P* = 0.067).

### Biomarkers levels across diagnosis groups

**Fig. 1:**
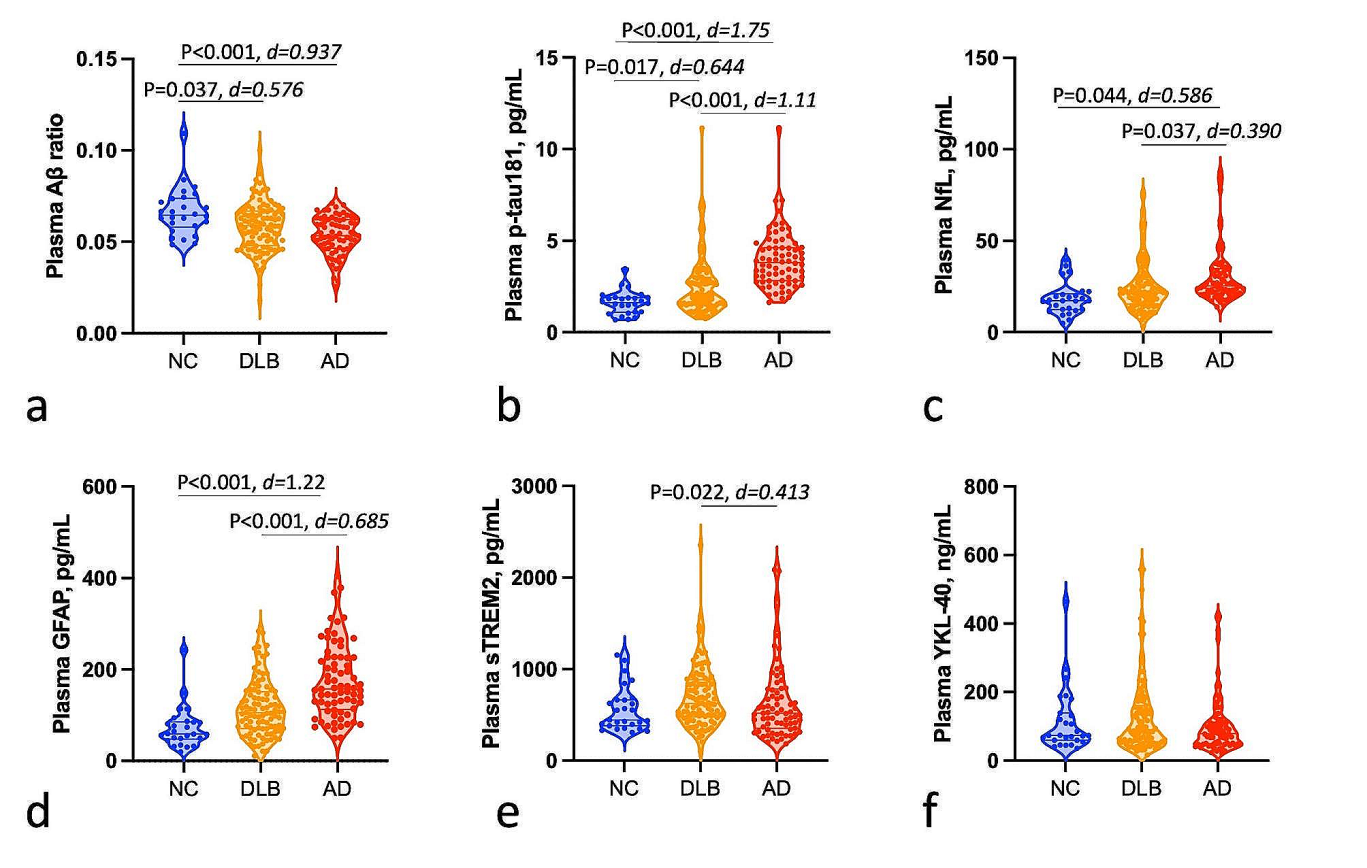
Plasma biomarkers levels across diagnosis groups. Plasma biomarkers levels across diagnosis groups including **a**, Aβ ratio; **b**, p-tau181; **c**, NfL; **d**, GFAP; **e**, sTREM2; and **f**, YKL-40. P-values were obtained through one-way ANCOVA followed by *post hoc* Tukey’s test, adjusting for multiple comparisons. Significant differences (*P* < 0.05) are reported. The effect size was determined using Cohen’s *d*. Boxplots display the median, IQR, and value for all participants

Plasma biomarker levels are displayed in Fig. 1. Patients with DLB displayed lower levels of plasma Aβ ratio (*P* = 0.037, *d* = 0.576) and higher p-tau181 (*P* = 0.017, *d* = 0.644) and a tendency to higher GFAP levels (*P* = 0.057, *d* = 0.057), compared to NC, after adjustment for age and sex. Additionally, patients with DLB displayed significantly lower levels of plasma p-tau181 (*P* < 0.001, *d* = 1.11), NfL (*P* = 0.037, *d* = 0.390), and GFAP (*P* < 0.001, *d* = 0.685) compared with AD patients. DLB patients had higher levels of plasma sTREM2 compared with AD patients (*P* = 0.022, *d* = 0.413). No difference was observed in plasma levels for YKL-40 between diagnostic groups, with or without adjustment. Plasma Aβ ratio levels were lower and p-tau181, GFAP, and NfL levels all higher in AD patients compared with controls, but not plasma sTREM2 and YKL-40.

Looking at AD stages, plasma p-tau181 levels remained significantly higher in both AD-MCI and AD dementia groups compared with the DLB group (Supp. Figure 3). Plasma NfL and GFAP levels were higher and sTREM2 levels lower in the AD dementia group compared with the DLB groups, but did not differ between AD-MCI and DLB.

### DLB diagnostic performance

**Fig. 2:**
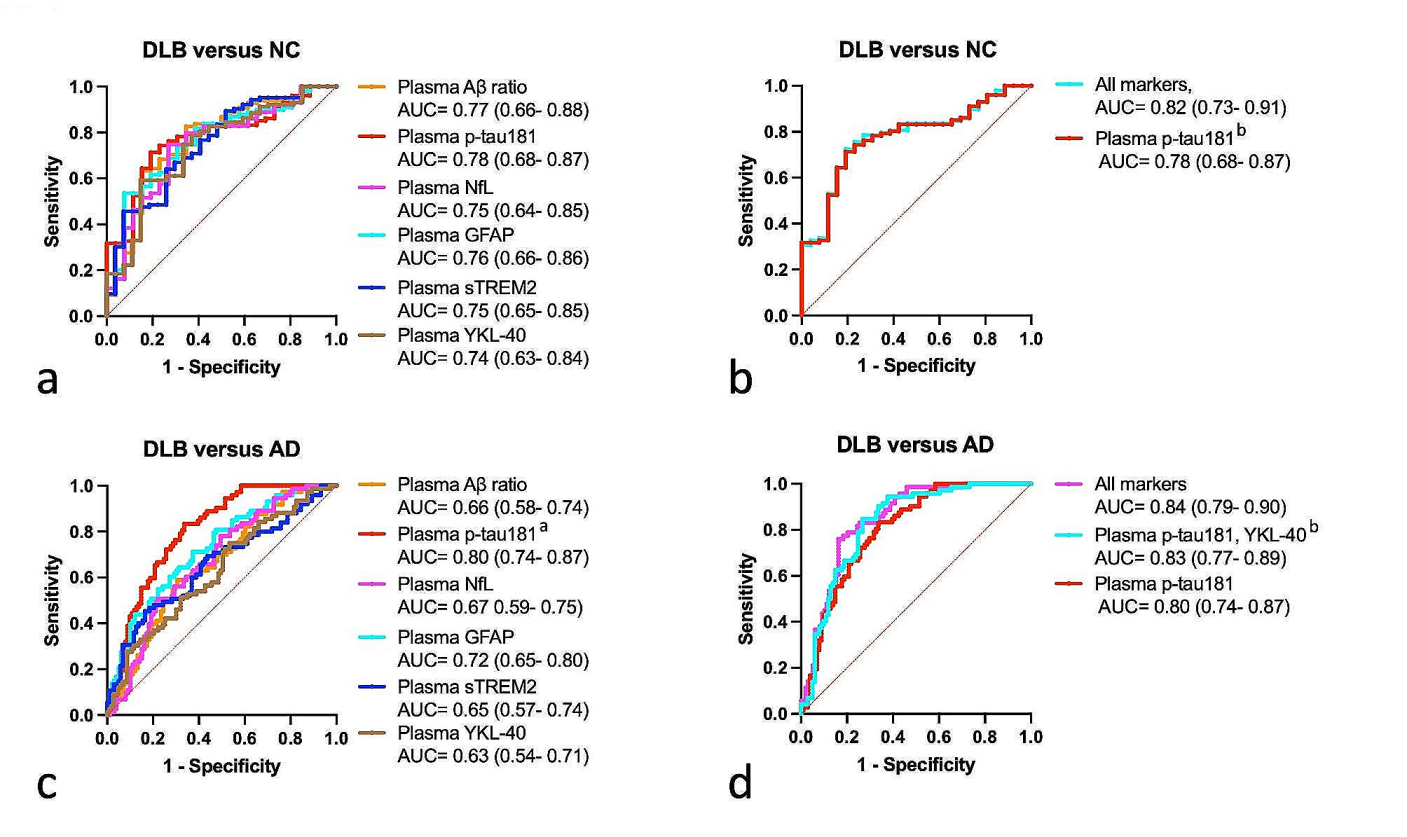
Plasma biomarkers performance to identify DLB. ROC analysis: **a**, to compare single biomarkers performance to discriminate between DLB patients and NC; **b**, to compare biomarkers combination to discriminate between DLB and NC; **c**, to compare single biomarkers performance to discriminate between DLB and AD patients; **d**, to compare biomarkers combination to discriminate between DLB and AD patients. ROC analysis results are presented as AUC (95% CI). Combinations of biomarkers were selected through binary logistic regression with backward stepwise elimination, including age and sex as constant variables. ^a^ model including p-tau181 outperformed all other models (∂AIC > 4), ^b^ no significant difference in the model’s fit with the All markers model (∂AIC < 4).

To differentiate DLB from controls, our plasma biomarkers yielded moderate AUCs from 0.74 to 0.78, without significant differences between biomarkers (Fig. 2a). Plasma p-tau181 yielded the highest AUC of 0.78 (95% CI 0.68–0.87). Combining biomarkers did not outperform p-tau-181 sole (Fig. 2b).

To differentiate DLB from AD, plasma p-tau181 yielded the highest AUC (0.80) as a standalone biomarker and outperformed the other biomarkers (∂AIC > 4, Fig. 2c). The optimal combination of markers was the association of plasma p-tau181 and YKL-40, that performed as well as the combination of all biomarkers (all biomarkers model, AUC = 0.84 versus plasma p-tau181 + plasma YKL-40, AUC = 0.83, ∂AIC < 4, Fig. 2d).

To differentiate AD from controls, plasma p-tau181 had the best performance as a standalone biomarker (AUC = 0.92) and association with other biomarkers did not improve diagnosis performance (Supp. Table 5).

The diagnosis performance of the plasma biomarkers used individually was overall similar when analyzing separately AD-MCI and AD dementia cases (Supp. Figure 3). The combination of plasma p-tau181 and YKL-40 had the best performance to differentiate DLB patients from AD-MCI (AUC = 0.86, ∂AIC > 4 versus all biomarkers model [AUC = 0.88] and p-tau181 alone [AUC = 0.80]), with the best trade-off between the goodness of fit and parsimony. To distinguish DLB from AD dementia, the association of plasma Aβ ratio, p-tau181, and NfL (AUC = 0.85) was not inferior to the all biomarkers model (AUC = 0.87, ∂AIC < 4).

#### Identification of amyloid copathology in DLB

**Fig. 3:**
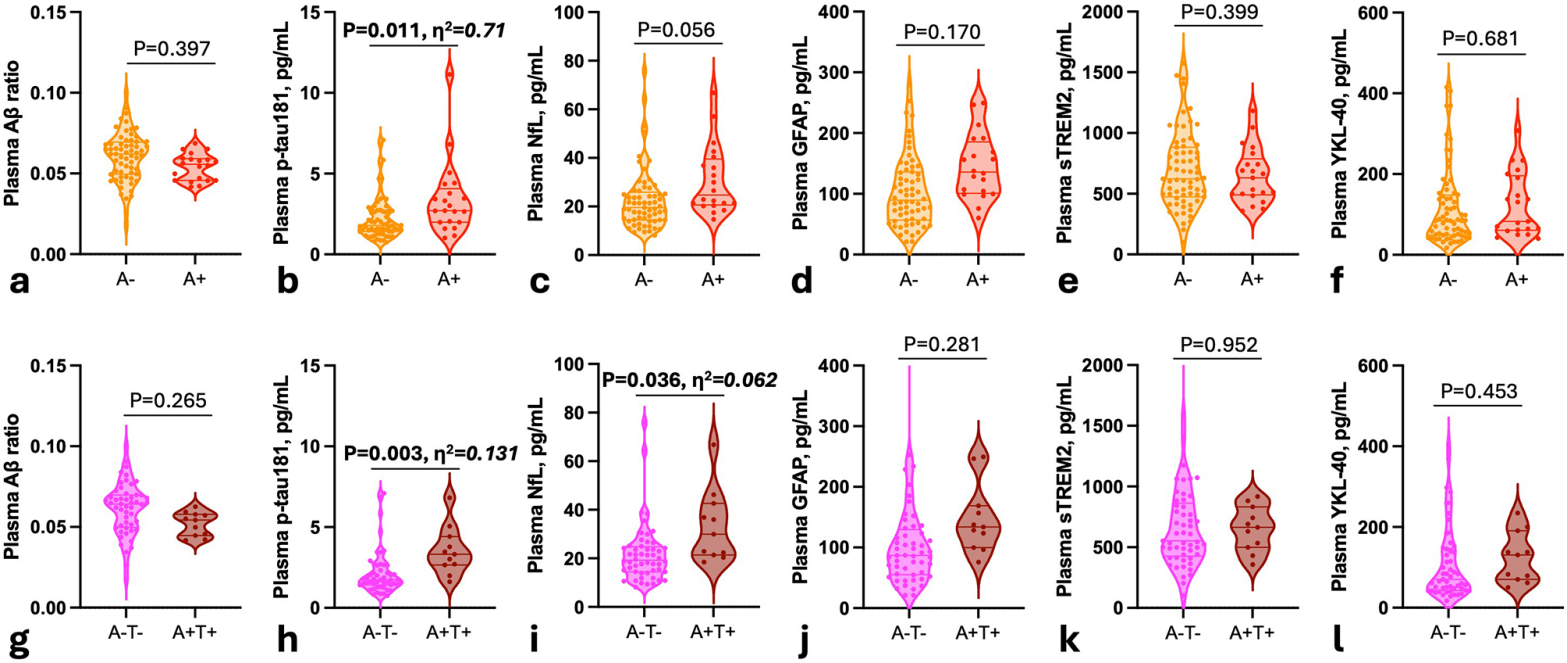
Plasma biomarkers levels in relation to amyloid pathology in DLB patients. Plasma biomarkers levels across amyloid-negative (A-) DLB and amyloid-positive (A+) DLB patients including **a**, Aβ ratio; **b**, p-tau181; **c**, NfL; **d**, GFAP; **e**, sTREM2; **f**, YKL-40; and across A-T- DLB and A + T + DLB patients including: **g**, Aβ ratio; **h**, p-tau181; **i**, NfL; **j**, GFAP; **k**, sTREM2; **l**, YKL-40. For biomarker levels comparison, P-values were obtained through one-way ANCOVA adjusting for multiple comparisons. Significant differences (*P* < 0.05) are reported in bold. The effect size was determined using η2. Boxplots display the median, IQR, and value for all participants

CSF analysis was available for 87% (90/104) of DLB patients (Table 1). According to the AT(N) classification, 24% of patients presented an AD CSF profile on the AD continuum, 12% being A + T- and 12% A + T+. A + DLB patients displayed higher concentrations of plasma p-tau 181 compared with A- DLB (*P* = 0.011, η2 = 0.71) after adjustment on age and sex (Fig. 3, a-f). A + T + patients displayed higher levels of plasma p-tau181 and NfL levels compared with A-T- DLB (respectively, *P* = 0.003, η2 = 0.131 and *P* = 0.036, η2 = 0.062, Fig. 3, g-l).

Plasma biomarkers identified A + DLB patients with overall moderate AUCs ranging from AUC = 0.64 to AUC = 0.75, as standalone biomarkers. Plasma p-tau181 displayed a higher AUC of 0.75, outperforming all other biomarkers (∂AIC > 4). The best combination of markers was the association of plasma p-tau181, GFAP, and NfL, yielding an AUC of 0.79, which was equivalent to the performance of the combination of all 6 plasma markers (AUC = 0.82, ∂AIC < 4 ). Plasma p-tau181 was outperformed by the combinations of all 6 biomarkers (AUC = 0.82 versus AUC = 0.75, ∂AIC = 5.5).

**Fig. 4:**
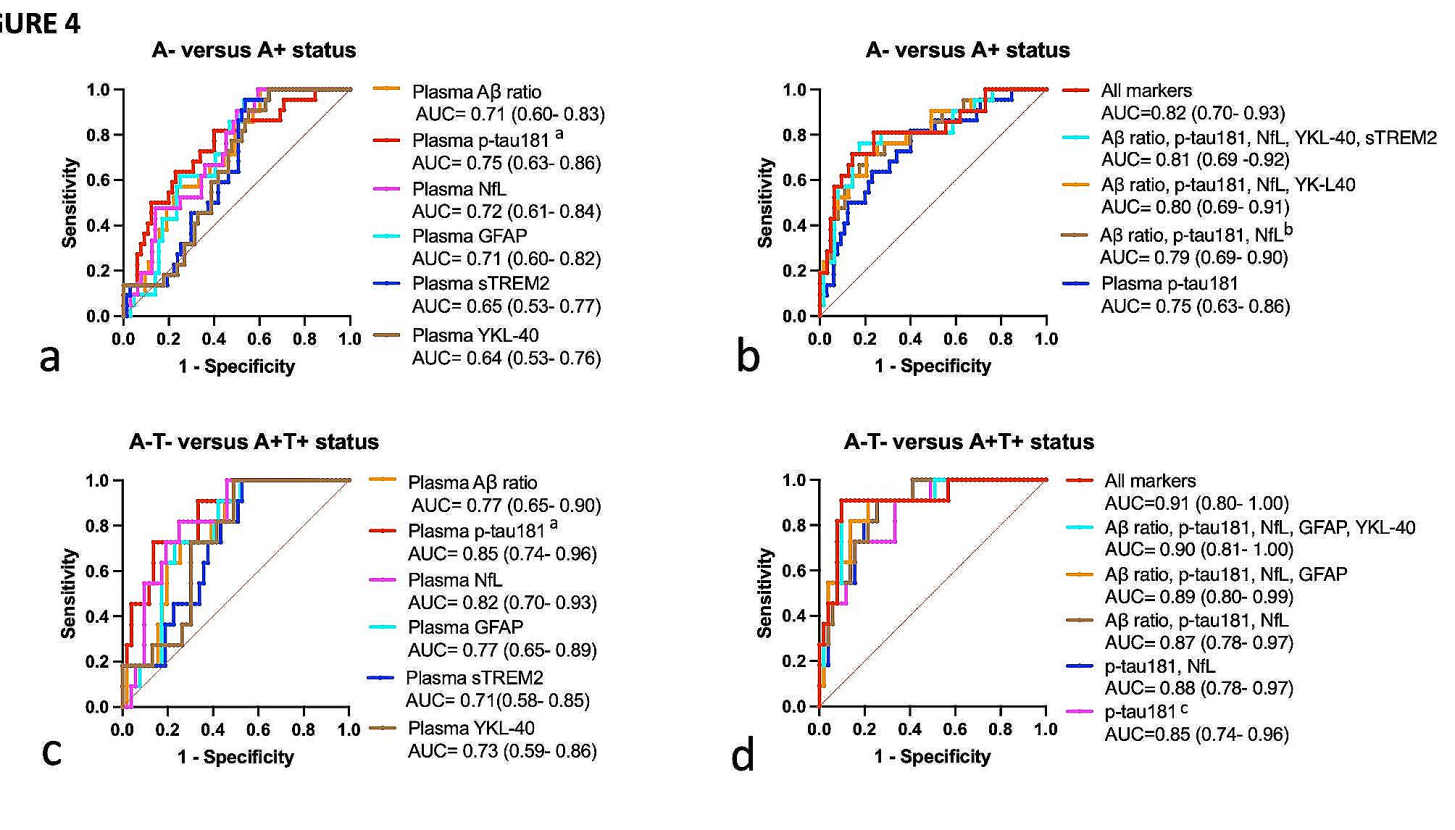
Plasma biomarkers performance for identification of amyloid copathology in DLB patients. ROC analysis: **a**, to compare single biomarkers performance to discriminate between A- and A + DLB patients; **b**, to compare biomarkers combination to discriminate between A- and A + DLB patients; **c**, to compare single biomarkers performance to discriminate between A-T- and A + T + DLB patients; **d**, to compare biomarkers combination to discriminate between A-T- and A + T + DLB patients. ROC analysis results are presented as AUC (95% CI). Combinations of biomarkers were selected through binary logistic regression with backward stepwise elimination, including age and sex as constant variables. ^a^the model including p-tau181 outperformed all other models (∂AIC > 4). ^b^the model associating plasma p-tau181, GFAP, and NfL was equivalent to the All markers models (∂AIC < 4).^c^the model including p-tau181 outperformed the All markers model (∂AIC > 4), with the best trade-off between parsimony and performance

Diagnosis performance of our plasma biomarkers was overall better in discriminating A + T + from A-T- DLB patients (AUC = 0.71–0.85, Fig. 4, c). Plasma p-tau181 displayed the highest AUC, of 0.85, outperforming all other biomarkers. Combining biomarkers (AUC = 0.87–0.91, Fig. 4, d) did not statistically outperform plasma p-tau181 sole (AUC = 0.85, ∂AIC  <4).

### Association with cognitive measurement

The associations of the plasma biomarkers with MMSE in diagnosis groups are presented in Supp. Table 6. In the DLB patients, we found higher plasma levels of p-tau181 levels were correlated with lower MMSE in unadjusted analysis (Spearman’s *r* = 0.231, *P* = 0.024). After adjustment on age, sex, and level of education, there remained no significant association (β=-0.176, *P* = 0.072). In the whole cohort, higher plasma p-tau181 and plasma GFAP levels were significantly associated with lower MMSE, after adjustment on age, sex, and level of education (respectively: β=-0.378 and β=-0.373, *P* < 0.001). In the AD group, higher plasma GFAP levels were correlated with lower MMSE in unadjusted analysis (*r* = -0.253, *P* = 0.032).

### Principal component analysis

**Fig. 5:**
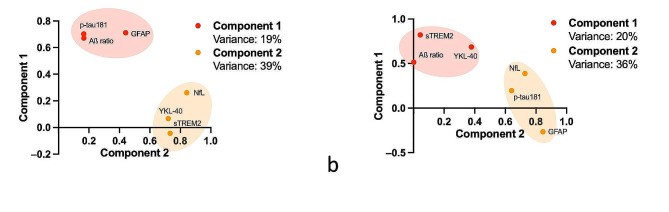
Principal component analysis of biomarker data in DLB and AD patients. **a**, Principal component analysis in DLB patients (*n* = 103). Component 1 associating plasma Aβ ratio, p-tau181, and GFAP explained 19% of the variance of the biomarkers data. Component 2 associating neuroinflammation sTREM2 and YKL-40 and axonal damage NfL makers explained 40% of variance. **b**, Principal component analysis in AD patients (*n* = 76). Component 1 associating plasma Aβ ratio, p-tau181, and GFAP explained 20% of the variance of the biomarkers data. Component 2 associating neuroinflammation sTREM2 and YKL-40 and axonal damage NfL markers explained 36% of variance

Lastly, we performed PCA to investigate the relationship between the different biomarkers in AD and DLB groups (Fig. 5). In DLB, we identified 2 principal components that explained 58% of the total variance in the dataset (Fig. 5, a). Component 1 accounted for 19% of the variance and was associated with plasma Aβ ratio, p-tau181, and GFAP. Component 2 captured 39% of the variance and was associated with neuroinflammatory markers sTREM2 and YKL-40 and axonal damage markers NfL. In the AD group, PCA analysis yielded two principal components as well (Fig. 5, b). First, a component 1 associated plasma Aβ ratio and neuroinflammatory markers sTREM2 and YKL-40, explaining 20% of the variance. A component 2 clustered plasma p-tau181, GFAP, and axonal damage markers NfL, capturing 36% of the variance.

## Discussion

In the present study, we report plasma biomarker modifications, including amyloid and tau, neurodegeneration, and neuroinflammation across a cohort of patients with probable DLB, compared with AD and controls. DLB patients displayed intermediate levels of plasma Aβ ratio, p-tau181and GFAP, falling in between control subjects and AD patients. Plasma p-tau181 was further altered in DLB patients with AD copathology. Subtle changes in plasma sTREM2 levels could be observed.

Plasma Aβ ratio, p-tau181, and GFAP levels were higher in DLB compared with NC but lower than those observed in the AD group. Those findings are in keeping with the previously published literature [25]. Plasma Aβ ratio was significantly lower in DLB patients compared with NC, even if the size of the effect was moderate compared with those of the decrease observed in the AD groups. Plasma Aβ42/40 has been reported to correlate with 18 F-florbetapir SUVR in DLB [17].

Regarding plasma p-tau, there is now significant evidence of its increase in DLB, already at the MCI stage [12, 14, 17]. The effect size difference was greater when comparing DLB and AD than between DLB and controls. Regarding diagnostic performance, p-tau181 had the highest performance in differentiating DLB from NC, and combining biomarkers did not improve diagnosis performance. To differentiate DLB from AD, p-tau181 also displayed the best performance, albeit moderate, in line with what has been reported in the literature [17, 25, 26]. Plasma GFAP displayed the largest effect size difference when comparing DLB to controls. It could reflect both copathology as GFAP has been demonstrated to be associated with Aβ mediated astrocytic reactivity, and general neurodegeneration [27]. In our study, it was not associated with CSF amyloid status, which could indicate Aβ-independent astrocytic activation or neurodegeneration. Indeed, there is emerging evidence that supports the existence of an astrocytic activation in DLB independently of amyloid pathology. Significant tracer uptake in 11 C-PK11195 microglial PET has been observed in DLB with no association with amyloid pathology [28]. Autopsy studies on DLB brains have demonstrated increased GFAP + astrocyte reactivity, in association with Lewy body pathology [29, 30]. If it is established that AD copathology has an important impact on the inflammatory signals detected in DLB, there is emerging evidence for specific astroglial processes related to Lewy body pathology, that could be picked up by plasma biomarkers.

No difference in plasma NfL levels was observed between NC and DLB, whereas there was a significant difference between DLB and AD groups. Previous findings regarding plasma NfL in DLB have been ambiguous, which may partly be because of small sample studies, discrepancies in design, and variability of cohorts, combining sometimes DLB with Parkinson’s disease dementia [31]. In several studies, plasma NfL was shown to reflect disease progression in later DLB stages as a non-specific marker of worse cognitive and clinical outcomes, as well as a reflection of amyloid copathology [32, 33].

In our cohort, no difference in levels of plasmaYKL-40 could be observed. Previous studies had reported no difference in CSF YKL-40 levels between DLB and controls [18]. In plasma, increased levels of YKL-40 have been described in a cohort of Lewy body dementia patients including DLB patients and Parkinson’s disease dementia patients [19]. Specific studies focusing on DLB cases will be needed to clearly state if CSF or plasma YKL-40 are consistently altered in DLB. In our work, the combination of plasma p-tau181 and YKL-40 levels increased performance to differentiate AD from DLB, which would still indicate an underlying glial process picked up by YKL-40. Plasma sTREM2 was higher in DLB compared with AD. High levels of CSF sTREM2 in DLB have already been reported [18]. Similar findings have been observed in PD brain, suggesting a reaction to alpha-synuclein deposition [34]. Plasma sTREM2 levels did not differ in A- and A + DLB subjects, suggesting that the observed increase in the DLB group is not related to AD pathology. However, it is still unclear if YKL-40 or sTREM2 plasma levels are the reflection of a central process, or of an associated peripherical immune dysregulation. In the brain, the expression and secretion of YKL-40 are attributed to astrocyte activation [35]. Brain-derived YKL-40 is hypothesized to then be released in the blood and contribute to plasma levels. Regarding sTREM2, while CSF levels are considered to reflect microglial inflammation, there is evidence that blood sTREM2 might reflect the activation of a wider range of myeloid cells [36]. In addition, there is growing evidence of altered peripherical immune response in DLB. High peripheral levels of cytokines and modified lymphocyte profile have been reported, at both MCI and dementia stages [37, 38]. While both the central and peripherical inflammation processes are likely key features of DLB, CSF and plasma neuroinflammation markers might likely provide different information.

Complementary biomarkers reflecting the other pathological mechanisms of DLB, such as, first and foremost, αsynuclein aggregation but also synaptic alterations, and mitochondrial dysfunction, would most likely contribute to diagnosis.

Amyloid deposition is common in dementia with Lewy bodies (DLB), ranging from 40 to 70% in neuropathological studies [39, 40]. Approximately half of patients with DLB demonstrate coexisting amyloid lesions, that impact clinical presentation and disease progression [41]. Plasma p-tau markers, including plasma p-tau181 and p-tau231, were shown to pick up amyloid pathology and correlate with cognitive decline, accordingly to CSF and PET markers [12, 14]. In our study, only plasma p-tau181 was significantly higher comparing the A + DLB patients compared with the A-. Comparing A + T + to A-T-, both plasma p-tau181(with a higher effect size) and NfL were increased. This suggests that plasma biomarkers display more significant abnormalities in DLB patients with AD copathology when abnormalities in CSF Aβ and p-tau are both established (A + T + stage). We did not observe a difference in CSF A + and A- DLB groups for plasma GFAP or Aβ ratio, conversely as what has already been reported [17]. We cannot exclude that our small samples of CSF amyloid-positive patients could have prevented us from measuring existing effects. However, combining p-tau181 to Aβ ratio and NfL significantly increased performance to identify A + patients, compared with the use of p-tau181 sole.

Additionally, we only found an association of MMSE with plasma-tau181 in DLB patients in unadjusted analysis, keeping in line with the reported poorer cognitive status of patients with amyloid copathology [12]. Thus, we add evidence to existing studies that p-tau181 is a valuable marker of AD co-pathology and expand on the potential of combining biomarkers.

Interestingly, our PCA analysis demonstrated different segregations of our biomarkers in AD and DLB groups, pointing towards differential underlying physiopathology. In DLB, axonal NfL and glial markers sTREM2 and YKL-40 clustered in a 1st component, suggesting neuroinflammation and axonal loss as driving most of the variance in the data set. Plasma, Aβ ratio, p-tau181, and GFAP clustered in an “amyloid component”, that can be hypothesized as reflecting amyloid copathology. Indeed, plasma GFAP has been reported to be an early and independent marker of astrocytosis reactive to Aβ pathology, associating closely with amyloid markers [27, 42]. In the AD group, plasma p-tau was associated with plasma GFAP and NfL in a first component explaining a higher part of the variance, in what could be identified as a tau and neurodegeneration component. In a 2nd component, plasma Aβ clustered with plasma glial markers. Studies on the longitudinal course of microglial activation along the AD continuum have reported an early peak at the MCI stage which could explain this segregation [43].

Our study included well-characterized DLB and AD patients and control subjects. It benefited from the use of biomarkers and reference diagnosis criteria. A strength is that our sample originated from clinical settings and thus brings ‘real-life’ evidence on the use of those novel biomarkers, compared to strictly selected research cohorts. Amyloid ratio, p-tau, NfL, and GFAP were measured with the established and highly accurate Simoa method.

This work does not go without limitations. CSF data about amyloid copathology was lacking for a small part of the cohort. We did not have available measurements of other p-tau isoforms than p-tau-181, notably of p-tau217 or p-tau231, which may be more sensitive and specific in early AD. There is still little evidence currently on possible differences in p-tau isoforms in diagnosis accuracy for DLB [12, 14, 15]. sTREM2 and YKL-40 levels were measured using Elisa, whereas all other biomarkers were measured using Simoa, which might have induced some variability. We had no available measurement of alpha-synuclein pathology, DLB patients being included on clinical diagnosis. However, the clinical criteria used have demonstrated high specificity [44]. Plasma biomarkers should also be investigated in comparison to other atypical parkinsonian syndromes and FTD syndromes, that constitute potential differential diagnoses for DLB. Finally, exploring the association of plasma biomarkers with the clinical features of the disease could further inform their use, and give some insight on the clinical heterogeneity observed within the DLB spectrum. Additionally, combining plasma biomarkers and other clinical and supportive biomarkers could provide a more accurate diagnosis and prognosis.

In conclusion, we found a specific pattern of impairment in plasma biomarkers of amyloid, tau axonal damage, and neuroinflammation in DLB patients. Plasma p-tau181 levels were elevated in DLB cases with AD comorbid pathology, which could have potential for selecting patients for Aβ targeting therapeutics. The diagnosis performance of our biomarkers for diagnosis remained moderate, underlying the need for further development of specific markers for synucleinopathies and DLB-specific biomarkers.

### Electronic supplementary material

Below is the link to the electronic supplementary material.

Supplementary Material 1

## Data Availability

Anonymized data will be shared upon reasonable request after approval by the local research ethics committee.

## Abbreviations

AD: Alzheimer’s disease
AIC: Akaike information criteria
AUC: area under the receiver operating characteristic curve
CI: confidence interval
DLB: dementia with Lewy bodies
NC: neurological controls
ROC curve: receiver operating characteristic curve
