# Supplementary material for: Plasma biomarkers of amyloid, tau, axonal, and neuroinflammation pathologies in dementia with Lewy bodies
Source: Alzheimers Res Ther. 2024 Jul 3;16:146. doi: 10.1186/s13195-024-01502-y (PMC11221164; doi:10.1186/s13195-024-01502-y)
Supplement: Supplementary file 1 — Supplementary Material 1 [file 13195_2024_1502_MOESM1_ESM.pdf]

Plasma biomarkers of amyloid, tau, axonal and neuroinflammation pathologies in dementia with Lewy bodies

Agathe Vrillon, Olivier Bousiges, Karl Götze, Catherine Demuynck, Candice Muller, Alix Ravier, Benoit Schorr, Nathalie Philippi, Claire Hourregue, Emmanuel Cognat, Julien Dumurgier, Matthieu Lilamand, Benjamin Cretin, Frederic Blanc, Claire Paquet.

Supplementaries

Supplementary Figure 1, Sensitivity analysis including only A+T+ AD subjects for plasma biomarkers levels and diagnosis performance

Supplementary Figure 2, Correlation between plasma biomarkers in the whole cohort and in the diagnosis subgroups

Supplementary Figure 3, Plasma biomarkers levels and their diagnosis performance, including AD-MCI and AD dementia groups

Supplementary Table 1, CSF AD biomarkers measurements methods and cut-offs for Strasbourg and Paris cohorts

Supplementary Table 2, Cohort characteristics and plasma biomarkers levels by center

Supplementary Table 3, Cohort characteristics and plasma biomarkers levels including AD-MCI and AD dementia groups

Supplementary Table 4, Association of plasma biomarkers with age, sex, and APOE ε4 carriership in the whole cohort and diagnosis subgroups

Supplementary Table 5, Plasma biomarkers performance for diagnosis

Supplementary Table 6, Association of MMSE with plasma biomarkers levels in the whole cohort and in the diagnosis subgroups

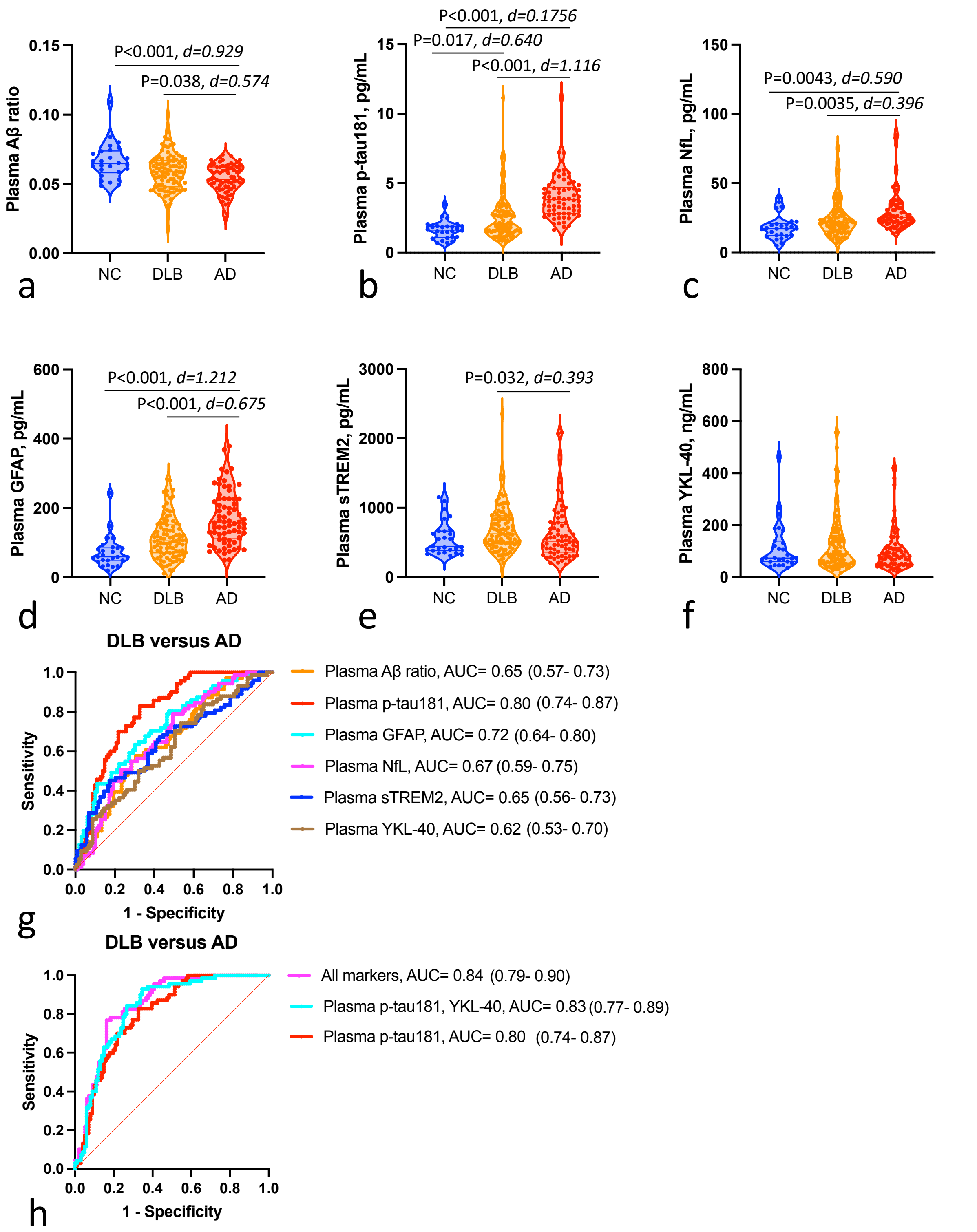

Supplementary Figure 1, Sensitivity analysis including only A+T+ AD subjects for plasma biomarkers levels and diagnosis performance

Plasma biomarkers levels across diagnosis groups including **a**, Plasma Aβ ratio; **b,** plasma p-tau181; **c**, plasma NfL; **d,** plasma GFAP; **e,** plasma sTREM2; and **f**, plasma YKL-40. P-values were obtained through one-way ANCOVA followed by post hoc Tukey‘s test, adjusting for

multiple comparisons. Significant differences ($P<0.05$) are reported. The effect size was determined using Cohen’s d. Boxplots display the median, IQR, and individual points for all participants.

ROC analysis: **g,** to compare single biomarkers performance to discriminate between DLB and AD patients; **h,** to compare biomarkers combination to discriminate between DLB and AD patients

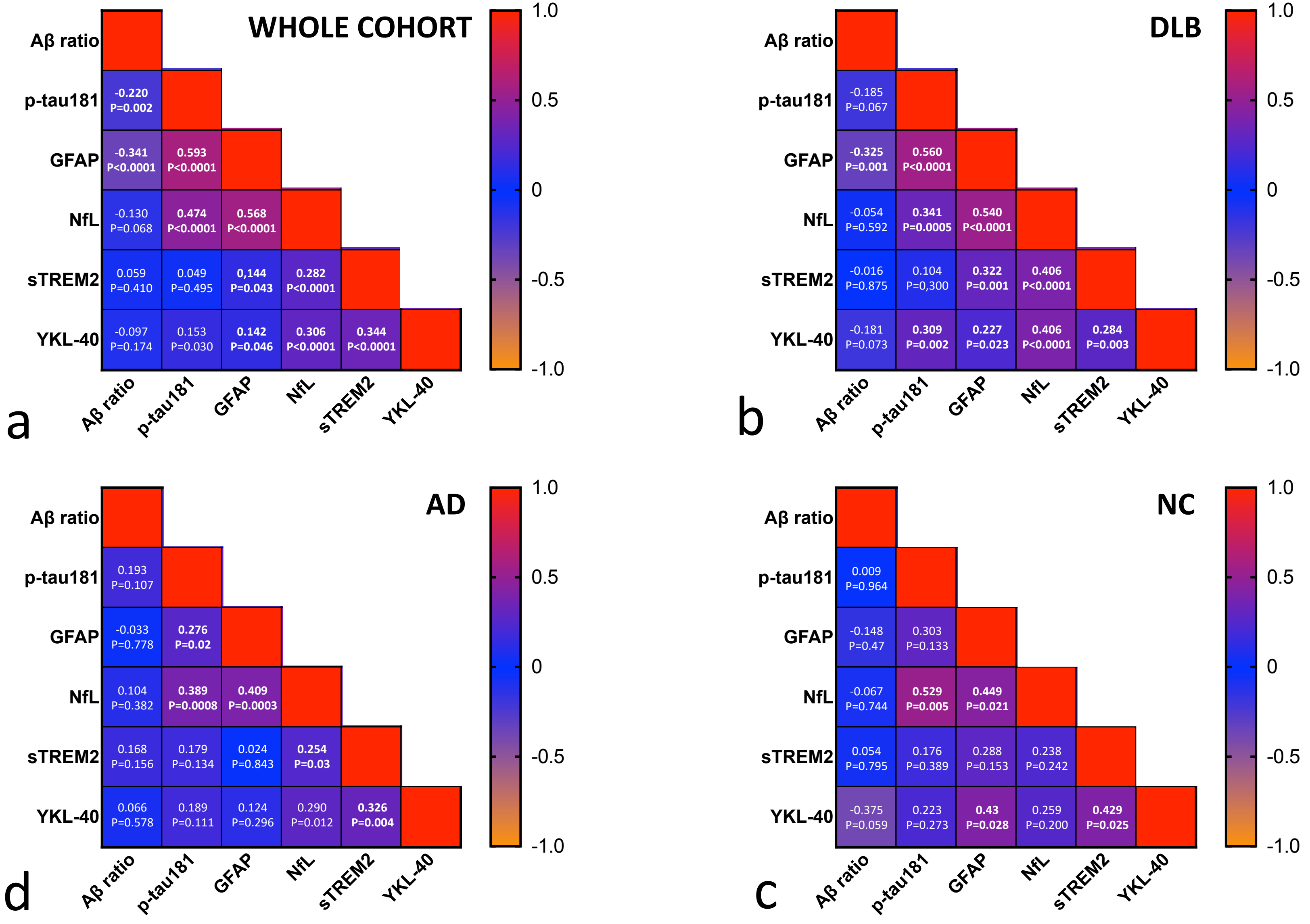
WHOLE COHORT
a
Aβ ratio
p-tau181
GFAP
NfL
sTREM2
YKL-40
-0.220 P=0.002
-0.341 P<0.0001
0.593 P<0.0001
-0.130 P=0.068
0.474 P<0.0001
0.568 P<0.0001
0.059 P=0.410
0.049 P=0.495
0,144 P=0.043
0.282 P<0.0001
-0.097 P=0.174
0.153 P=0.030
0.142 P=0.046
0.306 P<0.0001
0.344 P<0.0001
1.0
0.5
0
-0.5
-1.0
DLB
b
-0.185 P=0.067
-0.325 P=0.001
0.560 P<0.0001
-0.054 P=0.592
0.341 P=0.0005
0.540 P<0.0001
-0.016 P=0.875
0.104 P=0,300
0.322 P=0.001
0.406 P<0.0001
-0.181 P=0.073
0.309 P=0.002
0.227 P=0.023
0.406 P<0.0001
0.284 P=0.003
AD
d
0.193 P=0.107
-0.033 P=0.778
0.276 P=0.02
0.104 P=0.382
0.389 P=0.0008
0.409 P=0.0003
0.168 P=0.156
0.179 P=0.134
0.024 P=0.843
0.254 P=0.03
0.066 P=0.578
0.189 P=0.111
0.124 P=0.296
0.290 P=0.012
0.326 P=0.004
NC
c
0.009 P=0.964
-0.148 P=0.47
0.303 P=0.133
-0.067 P=0.744
0.529 P=0.005
0.449 P=0.021
0.054 P=0.795
0.176 P=0.389
0.288 P=0.153
0.238 P=0.242
-0.375 P=0.059
0.223 P=0.273
0.43 P=0.028
0.259 P=0.200
0.429 P=0.025

Supplementary Figure 2, Correlation between plasma biomarkers in the whole cohort and in the diagnosis subgroups

Correlation of plasma biomarkers in a, the whole cohort; b, DLB group; c, AD group; d, neurological controls. Correlations were computed with Spearman's correlation. Results are displayed as rho, P-value. Significant correlations ($P<0.05$) are indicated in bold.

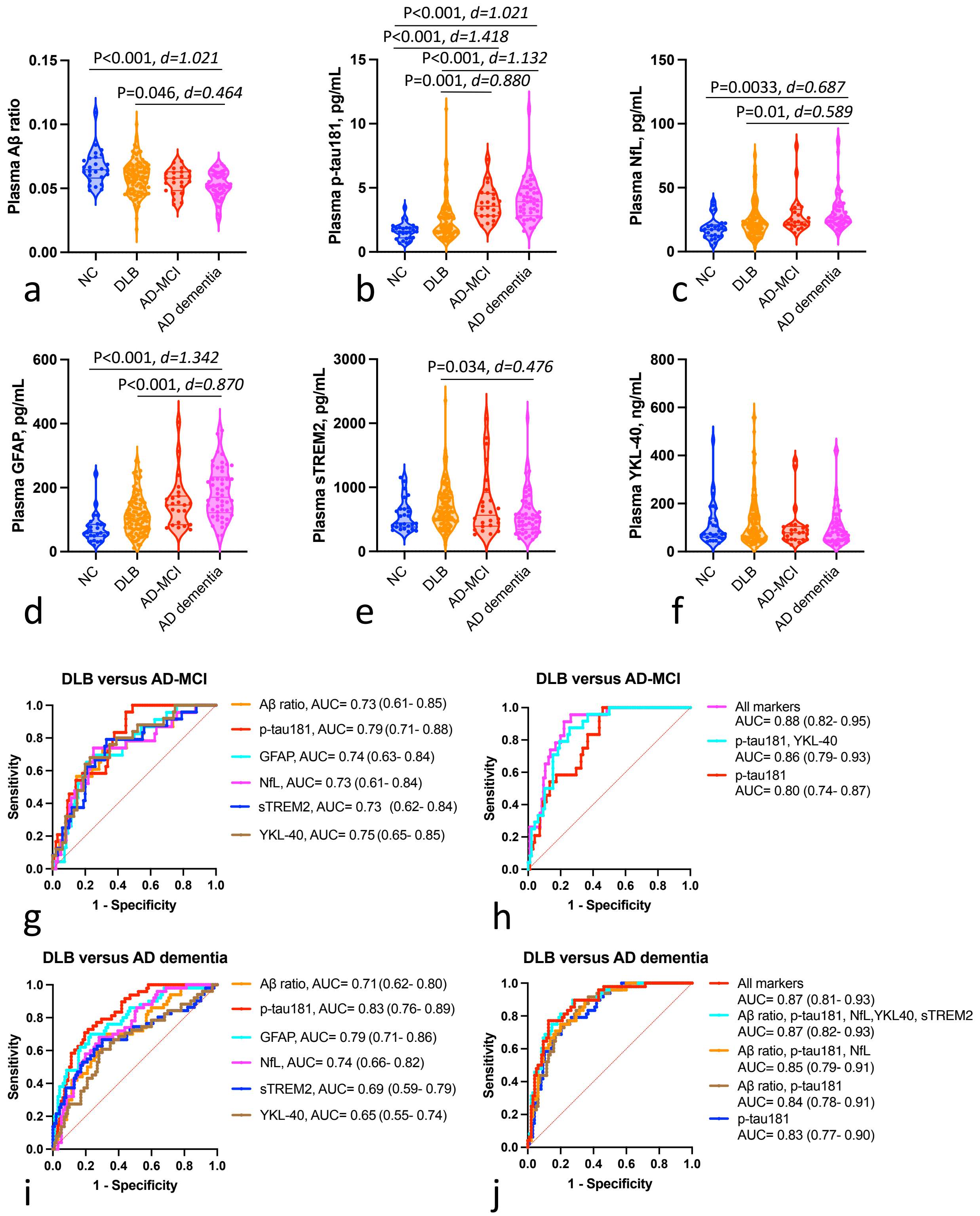

Supplementary Figure 3, Plasma biomarkers levels and their diagnosis performance, including AD-MCI and AD dementia groups

Plasma biomarkers levels across diagnosis groups including **a**, Plasma Aβ ratio; **b,** plasma p-tau181; **c**, plasma NfL; **d,** plasma GFAP; **e,** plasma sTREM2; and **f**, plasma YKL-40. P-values

were obtained through one-way ANCOVA followed by post hoc Tukey‘s test, adjusting for multiple comparisons. Significant differences ($P<0.05$) are reported. The effect size was determined using Cohen’s *d*. Boxplots display the median, IQR, and individual points for all participants.

ROC analysis: **g,** to compare single biomarkers performance to discriminate between DLB and AD-MCI patients; **h,** to compare biomarkers combination to discriminate between DLB and AD-MCI patients; **i,** to compare single biomarkers performance to discriminate between DLB and AD dementia patients; **j,** to compare biomarkers combination to discriminate between DLB and AD dementia patients. ROC analysis results are presented as AUC (95% CI). Combinations of biomarkers were selected through binary logistic regression with backward stepwise elimination, including age and sex as constant variables.

**Assay**	**Aβ 42**	**Aβ42/ Aβ40 ratio**	**P-tau**	**T-tau**	**References for used cut-offs**
			**Strasbourg Cohort**		
**Innotest Fujirebio ® n= 58**	<500 pg/mL	>0.1	<60 pg/mL	<500 pg/mL	Manufacturer recommendations
**Lumipulse® n=3**	<600 pg/mL	>0.05	<75 pg/mL	<500 pg/mL	Aβ42, Aβ42/Aβ40 ratio cut-offs: manufacturer recommendations (Ref 00008, 02/13/2019)<br>p-tau and t-tau cut-offs: derived from the centre's population
			**Paris Cohort**		
**Innotest Fujirebio® 1 n=4**	<815 pg/mL	>0.064	<58 pg/mL	<300 pg/mL	Published in Dumurgier J, et al. Intersite variability of CSF Alzheimer's disease biomarkers in clinical setting. *Alzheimers Dement.* 2013
**Innotest Fujirebio® 2 n=26**	<730 pg/mL	>0.076	<58 pg/mL	<340 pg/mL	Manufacturer recommendations
**Roche Elecsy® (Aβ42, p-tau, t-tau) / Innotest Fujirebio® (Aβ40) n=101**	>850 pg/mL	>0.083	<22 pg/mL	<225 pg/mL	Roche®: derived from the centre's population, published previously in Regy et al, ART 2023; Lilamand et al, The Journals of Gerontology: Series A 2023.<br>Innotest Fujirebio®: manufacturer recommendations

Supplementary Figure 1, CSF AD biomarkers measurements methods and cut-offs for Strasbourg and Paris cohorts

	PARIS Lariboisière			STRASBOURG CMRR		
	**NC**	**AD**	**DLB**	**NC**	**AD**	**DLB**
**Total n=207**	n=19	n=71	n=56	n=8	n=5	n=48
Age, years	61.3 [8.60]	72.0 [12.6]	72.2 [7.47]	60.5 [8.50]	77.0 [2.00]	68.0 [12.8]
Sex, male	30% (6)	39% (28)	69% (38)	25% (2)	40% (2)	46% (22)
APOE ε4 carriership*	35% (11/17)	70% (45/64)	47% (15/32)	25% (2)	40% (2)	33% (16)
MMSE	28.5 [2.75]	19.0 [5.75]	24.0 [6.00]	28.5 [1.0]	22 [6.00]	27.0 [5.00]
Level of education, years	15 [3.00]	9 [6.00]	11.0 [6.00]	15 [3.00]	11 [1.00]	11 [6.00]
**Plasma biomarkers levels**						
Plasma Aβ ratio	0.0646 [0.0101]	0.0587 [0.0151]	0.0530 [0.0151]	0.0637 [0.0233]	0.0591 [0.00873]	0.0592 [0.0201]
Plasma GFAP, pg/mL	1.51 [0.847]	1.87 [1.56]	3.85 [1.80]	1.79 [0.253]	3.18 [1.25]	2.10 [1.45]
Plasma NfL, pg/mL	69.1 [34.8]	105 [75.9]	162 [99.2]	57.1 [27.9]	91.4 [26.6]	114 [93.3]
Plasma p-tau181, pg/mL	15.1 [8.84]	21.5 [13.5]	25.3 [13.4]	18.8 [3.54]	26.4 [5.08]	21.5 [12.7]
Plasma sTREM2, pg/mL	568 [288]	539 [417]	510 [355]	418 [162]	1111 [470]	729 [432]
Plasma YKL-40, ng/mL	74.6 [95.7]	83.6 [106]	77.5 [66.7]	70.5 [51.4]	89.6 [26.3]	96.4 [101]

Supplementary Table 2, Cohort characteristics and plasma biomarkers levels presented by center

Continuous variables are presented as median [IQR] and categorical data as number (%).

Abbreviations: AD, Alzheimer disease; AUC, area under the curve; DLB, dementia with Lewy bodies; GFAP, glial fibrillary acidic protein; NC, neurological controls; MMSE, mini mental state examination; NfL, neurofilament light chain.

	NC	DLB	AD MCI	AD dementia	P-value
**Total n=207**	n=27	n=104	n=25	n=51	
Age, year	61.3 [10.0]	71.2 [11.4]	76.7 [7.9]	69.1 [14.8]	**<0.001**[a]
Sex, male	41% (11)	59% (61)	56% (14)	33% (17)	[b]**0.018**
APOE ε4 carriership	32% (8/17)	39% (31/79)	57% (13/23)	67% (34/46)	**<0.001**[b]
MMSE	28.5 [3.00]	25 [5.75]	26 [4]	18 [4]	**<0.001**[a]
Level of education, year	15.0 [5.0]	11.0 [6.0]	15.0 [4.0]	9 [4.5]	**<0.001**[a]
**Plasma biomarkers levels**					
Plasma Aβ40/Aβ42 ratio	0.0646 [0.0140]	0.0589 [0.0181]	0.0579 [0.0146]	0.0524 [0.0151]	**<0.001**
Plasma GFAP, pg/mL	59.4 [35.3]	107 [81.5]	146.0 [89.4]	169.5 [111.7]	**<0.001**
Plasma NfL, pg/mL	17.2 [7.95]	21.5 [14.0]	23.2 [12.4]	26.6 [13.2]	**<0.001**
Plasma p-tau181, pg/mL	1.62 [0.788]	2.03 [1.52]	3.57 [1.76]	3.92 [2.12]	**<0.001**
Plasma sTREM2, pg/mL	444 [274]	629 [397]	563.9 [527.3]	517.7 [396.5]	0.054
Plasma YKL-40, ng/mL	73.7 [75.3]	87.7 [103]	78.38 [54.10]	79.97 [79.40]	0.068

Supplementary Table 3. Cohort characteristics and plasma biomarkers levels including AD-MCI and AD dementia groups

Continuous variables are presented as median [IQR] and categorical data as number (%).

[a] Age, MMSE scores and level of education were compared between groups using Kruskall-Wallis test.[b] APOE ε4 carriership frequency was compared between groups using Chi2 test. *In-between groups comparison of plasma biomarkers levels was performed using one-way ANCOVA adjusted on age and sex.

		Age		Sex		ApoE4 status	
		Unadjusted	Adjusted on sex and ApoE4 status	Unadjusted	Adjusted on age and ApoE4 status	Unadjusted	Adjusted on age and sex
Plasma Aβ ratio	Whole cohort	r=-0.131, P= 0.066	β=-0.042, P=0.593	P=0.961	β=0.020, P=0.803	P=0.002	β=-0.228, P=0.004
	DLB	r=-0.133, P=0.190	β=-0.098, P=0.405	P=0.694	β=0.008, P=0.942	P=0.137	β=-0.142, P=0.233
	AD	r=0.215, P=0.068	β=0.263, P=0.037	P=0.485	β=0.046, P=0.711	P=0.076	β=0.250, P=0.042
	NC	r=-0.126, P=0.538	β=-0.190, P=0.852	P=0.087	β=0.200, P=0.404	P=0.653	β=-0.174, P=0.434
Plasma p-tau181	Whole cohort	r=0.254, P<0.001	β=0.261, P<0.001	P=0.913	β=0.039, P=0.598	P<0.001	β=0.220, P=0.004
	DLB	r=0.267, P=0.007	β=0.247, P=0.030	P=0.311	β=0.096, P=0.393	P=0.079	β=0.183, P=0.106
	AD	r=-0.113, P=0.345	β=-0.068, P=0.601	P=0.399	β=0.156, P=0.231	P=0.853	β=-0.008, P=0.950
	NC	r=0.325, P=0.106	β=0.321, P=0.173	P=1.000	β=0.024, P=0.917	P=0.787	β=0.068, P=0.754
Plasma GFAP	Whole cohort	r=0.393, P<0.001	β=0.464, P<0.001	P=0.011	β=0.258, P<0.001	P=0.013	β=0.091, P=0.181
	DLB	r=0.541, P<0.001	β=0.521, P<0.001	P=0.012	β=0.259, P=0.008	P=0.214	β=0.009, P=0.927
	AD	r=0.052, P=0.664	β=0.125, P=0.322	P=0.095	β=0.291, P=0.023	P=0.676	β=-0.092, P=0.455
	NC	r=0.591, P=0.001	β=0.609, P=0.004	P=0.878	β=0.063, P=0.745	P=0.569	β=0.172, P=0.344
Plasma NfL	Whole cohort	r=0.485, P<0.001	β=0.538, P<0.001	P=0.614	β=0.073, P=0.278	P=0.054	β=0.055, P=0.412
	DLB	r=0.422, P<0.001	β=0.490, P<0.001	P=0.683	β=0.106, P=0.302	P=0.867	β=-0.087, P=0.401
	AD	r=0.377, P=0.001	β=0.401, P=0.001	P=0.485	β=0.028, P=0.812	P=0.264	β=0.078, P=0.507
	NC	r=0.649, P<0.001	β=0.630, P=0.003	P=0.357	β=0.064, P=0.730	P=0.928	β=0.009, P=0.957
Plasma sTREM2	Whole cohort	r=0.287, P<0.001	β=0.310, P<0.001	P=0.610	β=0.042, P=0.578	P=0.267	β=0.165, P=0.028
	DLB	r=0.137, P=0.166	β=0.180, P=0.103	P=0.049	β=0.269, P=0.016	P=0.361	β=-0.179, P=0.108
	AD	r=0.401, P<0.001	β=0.386, P=0.001	P=0.063	β=0.158, P=0.176	P=0.765	β=-0.020, P=0.858
	NC	r=0.311, P=0.114	β=0.342, P=0.113	P=0.544	β=0.076, P=0.717	P=0.124	β=-0.326, P=0.110
Plasma YKL-40	Whole cohort	r=0.315, P<0.001	β=0.236, P=0.002	P<0.001	β=0.198, P=0.008	P=0.122	β=-0.092, P=0.215
	DLB	r=0.333, P<0.001	β=0.264, P=0.019	P=0.072	β=0.149, P=0.182	P=0.864	β=-0.068, P=0.544
	AD	r=0.319, P=0.005	β=0.274, P=0.020	P=0.003	β=0.281, P=0.018	P=0.923	β=-0.022, P=0.844
	NC	r=0.239, P=0.230	β=0.191, P=0.389	P=0.368	β=0.149, P=0.501	P=0.238	β=-0.201, P=0.340

Supplementary Table 4, Association of plasma biomarkers with age, sex and APOE ε4 carriership in the whole cohort and diagnosis subgroups.

Unadjusted associations of plasma biomarkers were studied with Spearman correlations for age and with Welchs-test for sex and APOE ε4 carriership. Adjusted analysis was performed using linear regression adjusting for age, sex, and APOE ε4 carriership. Standardized regression coefficient (β) estimates and p-values are displayed for adjusted analysis. Significant associations or correlations are displayed in bold.

	DLB versus NC		DLB versus AD		DLB versus AD MCI		DLB versus AD dementia		AD versus NC		A+ versus A- (whole cohort)	
	AUC	95% CI	AUC	95% CI	AUC	95% CI	AUC	95% CI	AUC	95% CI	AUC	95% CI
Plasma Aβ ratio	0.77	0.66- 0.88	0.66	0.58- 0.73	0.73	0.61-0.85	0.70	0.62-0.80	0.87	0.79- 0.96	0.72	0.64- 0.79
Plasma p-tau181	0.78	0.68- 0.87	0.80[a]	0.74- 0.87	0.79	0.71-0.88	0.83	0.76-0.89	0.97[a]	0.93- 1.00	0.84[*]	0.78- 0.90
Plasma NfL	0.75	0.64- 0.85	0.67	0.59- 0.75	0.73	0.61-0.84	0.74	0.66-0.82	0.85	0.75- 0.95	0.74	0.67- 0.81
Plasma GFAP	0.76	0.66- 0.86	0.72	0.65- 0.80	0.74	0.63-0.84	0.79	0.71-0..86	0.91	0.84- 0.97	0.78	0.72- 0.85
Plasma sTREM2	0.75	0.65- 0.85	0.65	0.57- 0.74	0.73	0.62-0.84	0.69	0.59-0.79	0.78	0.67- 0.88	0.67	0.59- 0.74
Plasma YKL-40	0.74	0.63- 0.84	0.63	0.54- 0.71	0.75	0.65-0.85	0.65	0.55-0.74	0.79	0.70- 0.88	0.65	0.57- 0.72

Supplementary Table 4, Plasma biomarkers performance for diagnosis

Areas under the curve (AUC) were calculated using ROC analysis and binary logistic regression models, including the biomarker, age, and sex, to evaluate the performance of each biomarker for distinguishing between diagnostic groups and to identify amyloid positivity. Akaike information criterion (AIC) was calculated for each logistic regression model. [a]the model including p-tau181 outperformed all other models (∂AIC>4)Abbreviations: AD, Alzheimer's disease; AUC, area under the curve; DLB, dementia with Lewy bodies; GFAP, glial fibrillary acidic protein; NC, neurological controls; NfL, neurofilament light chain; 95% CI, 95% confidence interval

	**Whole cohort**		**NC**		**AD**		**DLB**	
	Unadjusted	Adjusted on sex, age, LoE	Unadjusted	Adjusted on sex, age, LoE	Unadjusted	Adjusted on sex, age, LoE	Unadjusted	Adjusted on sex, age, LoE
Plasma Aβ ratio	**r=-0.194 (P=0.007)**	β=0.092 (-0.224-0.040), P=0.171	r=-0.049 (P=0.816)	β=0.016 (-0.490-0.459), P=0.946	r=-0.071 (P=0.554)	β=0.013 (-0.216-0.242), P=0.908	r=0.059 (P=0.573)	β=-0.116 (-0.073-0.305), P=0.226
Plasma p-tau181	**r=0.461 (P< .001)**	β=**-0.378 (-0.253—-0.503), P< .001**	r=0.119 (P=0.570)	β=-0.065 (-0.453-0.583), P=0.796	r=0.142 (P=0.239)	β=0.082 (-0.147-0.311), P=0.477	**r=-0.231 (P=0.024)**	β=-0.176 (-0.016-0.369), P=0.072
Plasma GFAP	**r=0.444 (P< .001)**	β=**-0.373 (-0.229—-0.517), P< .001**	r=0.365 (P=0.073)	β=-0.464 (-0.046-0.974), P=0.072	**r=-0.253 (P=0.032**)	β=0.214 (-0.016-0.444), P=0.067	r=-0.156 (P=0.134)	β=-0.049 (-0.188-0.286), P=0.681
Plasma NfL	**r=0.327 (P< .001)**	β=**-0.178 (-0.032—-0.325), P=0.017**	r=-0.010 (P=0.964)	β=0.087 (-0.638-0.463), P=0.744	r=0.161 (P=0.178)	β=0.207 (-0.0369-0.4500), P=0.095	r=-0.139 (P=0.183)	β=-0.040 (-0.170-0.250), P=0.705
Plasma sTREM2	r=-0.121 (P=0.090)	β=**-0.139 (-0.273—-0.0038), P=0.044**	**r=0.402 (P=0.042)**	β=-0.429 (-0.015-0.873), P=0.058	r=-0.208 (P=0.075)	β=-0.005 (-0.258-0.247), P=0.968	r=-0.136 (P=0.183)	β=0.170 (-0.365-0.025), P=0.087
Plasma YKL-40	r=0.010 (P=0.889)	β=0.094 (-0.231-0.042), P=0.175	r=0.350 (P=0.079)	β=-0.371 (-0.068-0.810), P=0.094	r=-0.016 (P=0.894)	β=0.089 (-0.155-0.332), P=0.468	r=-0.012 (P=0.909)	β=0.132 (-0.325-0.061), P=0.177

Supplementary Table 5, Association of MMSE with plasma biomarkers levels in the whole cohort and in the diagnosis subgroups. Unadjusted analysis was studied with Spearman's r correlations. Adjusted analysis on age, sex and level of education was performed using linear regression with post Hoc Tukey's adjusting for multiple comparisons. Standardised regression coefficient (β) estimates and p-values are displayed for adjusted analysis.

Abbreviations: AD, Alzheimer's disease; DLB, dementia with Lewy bodies; GFAP, glial fibrillary acidic protein; LoE, level of education; MMSE, mini mental state examination; NC, neurological controls; NfL, neurofilament light chain; p-tau181, tau phosphorylated at serine 181; sTREM-2, soluble triggering receptor expressed myeloid-
